# Mycoplasmas–Host Interaction: Mechanisms of Inflammation and Association with Cellular Transformation

**DOI:** 10.3390/microorganisms8091351

**Published:** 2020-09-04

**Authors:** Francesca Benedetti, Sabrina Curreli, Davide Zella

**Affiliations:** 1 Department of Biochemistry and Molecular Biology, Institute of Human Virology, School of Medicine, University of Maryland, Baltimore, MD 21201, USA; fbenedetti@ihv.umaryland.edu; 2 Department of Medicine, Institute of Human Virology, School of Medicine, University of Maryland, Baltimore, MD 21201, USA; SCurreli@ihv.umaryland.edu

**Keywords:** *Mycoplasma*, cancer, inflammation, molecular pathways, p53, PARP

## Abstract

Mycoplasmas are the smallest and simplest self-replicating prokaryotes. Located everywhere in nature, they are widespread as parasites of humans, mammals, reptiles, fish, arthropods, and plants. They usually exhibiting organ and tissue specificity. Mycoplasmas belong to the class named Mollicutes (mollis = soft and cutis = skin, in Latin), and their small size and absence of a cell wall contribute to distinguish them from other bacteria. *Mycoplasma* species are found both outside the cells as membrane surface parasites and inside the cells, where they become intracellular residents as “silent parasites”. In humans, some *Mycoplasma* species are found as commensal inhabitants, while others have a significant impact on the cellular metabolism and physiology. Mollicutes lack typical bacterial PAMPs (e.g., lipoteichoic acid, flagellin, and some lipopolysaccharides) and consequently the exact molecular mechanisms of Mycoplasmas’ recognition by the cells of the immune system is the subjects of several researches for its pathogenic implications. It is well known that several strains of *Mycoplasma* suppress the transcriptional activity of p53, resulting in reduced apoptosis of damaged cells. In addition, some Mycoplasmas were reported to have oncogenic potential since they demonstrated not just accumulation of abnormalities but also phenotypic changes of the cells. Aim of this review is to provide an update of the current literature that implicates Mycoplasmas in triggering inflammation and altering critical cellular pathways, thus providing a better insight into potential mechanisms of cellular transformation.

## 1. Mycoplasmas: Classification, Morphology, Genome Structure, and Organization

Mycoplasmas range from 0.1–0.3 μm in diameter and up to 98 μm in length and are the smallest and simplest self-replicating prokaryotes. Located everywhere in nature, they are widespread in humans, mammals, reptiles, fish, arthropods, and plants. They live on the mucous surface of the respiratory and urogenital tracts, in the eyes, in the alimentary canal, in the mammary glands and in the joints, usually exhibiting organ and tissue specificity [1]. Mycoplasmas belong to the class named Mollicutes (mollis = soft and cutis = skin, in Latin), and their small size and absence of a cell wall contribute to distinguish them from other bacteria [2].

One hypothesis (reductive or degenerative evolution) states that Mycoplasmas lost the cell wall and other biosynthetic pathways by adopting a parasitic lifestyle. According to this hypothesis, the parasitic way of life made disposable the presence of a cell wall. Consequently, Mycoplasmas progressively lost the genes necessary for the synthesis of the polymers necessary to build the cell wall. By living as parasites in the environment of their host, this development did not result in an evolutionary disadvantage. As a tradeoff, Mycoplasmas depends on their host for a number of essential nutritional requirements, and this has hampered their growth in culture and consequently a detailed study of their pathogenic determinants.

Mycoplasmas have a small circular double-stranded genome, variable among strains of the same species, ranging from less than 600 kb to 2200 kb, and they synthesize a relatively small number of proteins; thus, having limited metabolic capabilities. In fact, Mycoplasmas’ membrane is very simple, rigid, thin and resistant, composed of sterols (fatty acids, cholesterol, or complex lipids). The molecules are taken up from the surrounding environment and not synthesized by these microorganisms; their replication and survival depend on factors produced by the host or taken up by the growth medium [3].

*Mycoplasma* genome has a low guanine–cytosine (G + C) content and its variability is due to repetitive elements, consisting of segments of genes, different in size and number, or insertion sequence elements (IS) [4]. Their shape is controlled by the presence of a cytoskeleton that contributes also to the cell division (the reproduction occurs by binary fission) and to the motility of *Mycoplasma*. Mycoplasmas dominating shape is a sphere, but they can have small coccid bodies, swollen ring like forms, and filamentous-branched forms of variable length [2].

*Mycoplasma* species are found both outside the cells as membrane surface parasites and inside the cells, where they become intracellular residents as “silent parasites” [5]. Additional data showed the intracellular localization of *Mycoplasma fermentans* in cellular samples of AIDS patients [6] and that a *Mycoplasma* (named *Mycoplasma penetrans*) is capable of entry into many different human cells both in vivo and in vitro [7]. Recently, confocal micrographs demonstrated the ability of *Mycoplasma pneumonia* to bind and to internalize, depending on the cellular types [8,9].

Recognized as pathogens and co-factors in several diseases, Mycoplasmas generally cause chronic infections, and the identity and mechanisms of actions of most of their pathogenic determinants are not completely understood [10]. Mycoplasmas tend to colonize, damage, and invade the deep tissues as a result of mucosal surface disruption, local trauma, surgery, tissue necrosis, and impaired clearance of a sterile site. As they can grow in anaerobic environments, this may result in localized infections [11]. In fact, in a number of cases Mycoplasmas are considered causative agents for these localized infections, and the difficulty in their isolation and identification through laboratory practices likely renders these associations underestimated [12,13,14,15,16]. The recent addition of real time polymerase chain reaction (RT-PCR) with specific primers allowed the specific determination of the presence of Mycoplasmas in the site(s) of interest. For example, RT-PCR is the diagnostic method of choice for *Mycoplasma genitalium*, which is not detected on routine culture due to extremely slow growth. In other cases, for example with *Ureaplasma urealyticum* and *Mycoplasma hominis,* their fast growth allows the use of routine culture to determine their presence and in this case RT-PCR could be used as a confirmatory and faster assay [17].

The advancement in protein sequences techniques [18] have allowed the identification of potential determinants of Mycoplasmas’ pathogenicity both in humans (for example *Mycoplasma pneumoniae* [19,20], *Mycoplasma genitalium* [21,22], and *Mycoplasma fermentans* [23,24]) and in animals (*Mycoplasma mobile* in fish [25], *Mycoplasma hypopneumoniae,* and *Mycoplasma. flocculare* in swine [26,27]).

Consequently, several data indicate that the interactions of lipid proteins present on the membrane of Mycoplasmas interact with monocyte/macrophages modulating the immune response and sometimes resulting in immune system evasion [28,29,30].

## 2. Mycoplasmas and Inflammation

### 2.1. Mycoplasmas Causing Diseases in Humans

Whether attached to the surface of eukaryotic cells or upon invasion, some Mycoplasmas interfere and alter cellular pathways of the host cell, both at the regulation and/or functional level [28] (Table 1). To protect itself from such detrimental consequences, the host organism engages upon infection a series of responses that involves a number of signaling pathways, eventually resulting in the activation of both innate and acquired immunity, which elicit processes stimulating acute and chronic inflammation, respectively. In turn Mycoplasmas developed mechanisms to escape immune control, in such a way that they are able to colonize mucosal surfaces and invade different areas of the body. The outcome of this race between the host and the pathogen is determined by the efficiency and effective cooperation of the immune response, involving both components of the immune system, the humoral one and the cell-mediated one. Nonetheless, due to the delay between initial triggering and development of a full-scale response, Mycoplasmas often are able to adapt [31,32].

As mentioned above, *Mycoplasma* attaches to the outside cellular membrane, resulting in the interaction between certain bacterial proteins (lipoproteins (LPs)/lipopeptides or specific attachment organelles) on one hand, with specific cellular receptors on the surface of the target cells on the other hand. To this regard, a number of studies have identified several Mycoplasmas’ LPs that can interact with epithelial cells and leukocytes of the host organism [31,33,34]. When these bacterial proteins engage particular receptors expressed in immune cells (pattern-recognition receptors (PRR)), an inflammatory reaction ensues. More in detail, the pathogen-associated molecular pattern (PAMPs) are recognized by cells of the innate immune system through interaction with specialized PRRs—Toll-like receptors (TLRs) and nucleotide-binding oligomerization domain-containing protein (NOD)-like receptors. In general, TLRs are the first molecules to interact with PAMPs and subsequent to this event, the specificity of the immune response against a certain infectious agent is determined by the specific signaling pathway engaged by the interaction [35]. Moreover, some PRR can recognize certain endogenous signals (including of bacterial origin) originating upon tissue or cell damage events, and for this reason are named danger-associated molecular patterns (DAMPs) [34]. It is worth noting that the exact molecular mechanisms of recognition of Mollicutes by the immune system is the focus of active studies, because many classical bacterial PAMPs are indeed not expressed in certain Mycoplasmas (for example, lipoteichoic acid, flagellin, some lipopolysaccharides (LPS)).

Bacterial LPs bind TLRs 1, 2, 4, and 6 [36,37], and, the first lipopeptide expressed in Mycoplasmas demonstrated to bind TLRs was the macrophage-activating lipopeptide-2 (MALP-2) of *Mycoplasma fermentans.* Subsequently, triacylated or diacylated lipopeptides were shown to bind heterodimers of TLR 1/2 or TLR 2/6, respectively [38,39]. An in vivo confirmation of this set of events came with the observation that TLR2-knockout mice could not induce signaling mediated by MALP-2. Additionally, to underline the importance of this signaling pathway, binding of lipoprotein/lipopeptide with TLRs results in cellular activation and in the downstream expression of NF-κB. Some of the inflammatory diseases linked to infections by Mycoplasmas are mastitis, salpingitis, urethritis, arthritis atypical pneumonia, and bronchopulmonary dysplasia, which is particularly dangerous for newborns [40]. Such inflammation is elicited by the presence of specific immune mediators, released by target cells (epithelial cells and leukocytes) upon infection by Mycoplasmas. This event, in turn, promotes the expression of proinflammatory cytokines and chemokines, a subsequent activation of NF-κB, and migration of certain cells including granulocytes, macrophages, and lymphocytes, ultimately leading to their recruitment to the site of infection [41]. Among the most important pro-inflammatory cytokines and chemokines we note tumor necrosis factor alpha(TNF-α), interleukin-6 (IL-6), macrophage inflammatory protein-1β (MIP-1β), growth-regulated alpha protein (GRO-α), monocyte chemoattractant protein 1 (MCP-1), MIP-1α [42], C-X-C motif chemokine 13 (CXCL13), C-X-C motif chemokine 14 (CXL14), regulated on activation, normal T cell expressed and secreted (RANTES) [43], and MIP-2 [44]. Interestingly, individual lipopeptides (e.g., triacylated lipopeptides) isolated and purified from Mycoplasmas can promote leukocyte infiltration in the respiratory tract, indicating a putative action of these factors also in the absence of the whole *Mycoplasma* organism [45]. Finally, it is worth mentioning that TLRs biding leading to the activation of NF-κB may engage the antiapoptotic program in the cell, which may eventually result in a pro-cancer activity [46].

Several mechanisms are being employed by Mycoplasmas to escape the inflammatory immune response. For example, *Mycoplasma genitalium* is able establish chronic urogenital infections by (a) expression of two antigenic proteins associated with attachment (MgpB and MgpC variants) with different amino acid sequences, and (b) phase variation, during which *Mycoplasma* lose the ability to adhere to cultured cells and instead acquires the ability to bind to red blood cells (hemadsorption) [47]. In another example, resistance of *Mycoplasma pneumoniae*, a causative agent of respiratory infection, to in vitro killing by neutrophils has been demonstrated, For this purpose, *Mycoplasma pneumoniae* employs Mpn491, a secreted nuclease, as a mean for evading the killing mechanism of infiltrated neutrophils, production of inflammatory mediators, and induction of local and systemic antibodies [48].

In humans, some *Mycoplasma* species are found as commensal inhabitants, while others have a significant impact on the cellular metabolism and physiology. Consequently, infection of the host cells results in the production of reactive oxygen species (ROS). For example, *Mycoplasma pneumoniae* induces production of ROS following infection, and the over-expression of some cellular proteins, in particular, glucose-6-phosphate 1-dehydrogenase (G6PD), NADH dehydrogenase (ubiquinone) Fe-S protein 2, and ubiquinol-cytochrome *c* reductase complex core protein I mitochondrial precursor indicated their involvement in regulating cellular oxidative status. This in turn caused an increase in DNA damage [49].

Mycoplasmas can also be associated with infectious diseases and post-infection pathologies, and frequently persist as chronic, asymptomatic infections both in humans and animals [28]. In fact they can cause a wide variety of diseases, including genitourinary tract [50], joint infections [51,52], neurologic disorders [53,54], and acute respiratory illness [55].

*Mycoplasma* species relevant to the urogenital tract include *Mycoplasma hominis*, *Mycoplasma genitalium,* and *Ureaplasma urealyticum*. Thought once classified as Mycoplasmas, Ureaplasmas are now defined and differentiated from *Mycoplasma* species by their characteristic lysis of urea. The persistence of irritable bladder symptoms following a urinary tract infection is a challenging situation for clinicians, because of the need to identify the presence of possible pathogens for a correct diagnosis. While most uropathogenic organisms—especially those originating from feces—can be demonstrated on standard culture, *Mycoplasma* and *Ureaplasma* species present technical challenges for their isolation, as mentioned earlier. In addition, these organisms may be found in both asymptomatic [56] and symptomatic patients with sterile leukocyturia [57]. In women, pathological significance is differentiated from harmless colonization by the presence of clinical symptoms, though bacterial count in urine does not necessarily correlate with the amount of bacteria in the bladder wall. In fact, the presence of a significant number of these intracellular organisms may be demonstrated in the bladder wall in the absence of bacteriuria. Pyelonephritis is another condition associated with *Mycoplasma hominis* and *Ureaplasma urealyticum* [58]. It is hypothesized that these pathogens reached the renal pelvis from the lower urinary tract. Consequently, bacterial colonization of the upper urinary tract is not completely demonstrated in catheter urine from the bladder [59]. The so-called “non-chlamydial non-gonococcal urethritis due to *Mycoplasma* and *Ureaplasma* infection,” is frequently described in men. Urethritis in women have been associated with *Mycoplasma hominis*, *Ureaplasma urealyticum* [60], and *Mycoplasma genitalium* [61,62,63], but clear evidence of causative effect by these microorganisms is still lacking.

In addition to being associated to respiratory diseases, *Mycoplasma pneumonia* is also found in several extra-respiratory conditions without a previous, clinically evident respiratory episode [64]. Among these conditions, the most difficult to be diagnosed and treated are diseases affecting the nervous system, both the peripheral (PNS) and the central nervous system (CNS). *Mycoplasma pneumonia* positivity can be detected in 5–10% of patients presenting with acute, febrile CNS disease, and represent a medical emergency [65,66,67,68]. In some cases, *Mycoplasma pneumoniae*-related neuropathies can lead to death or to persistent neurologic problems [64,69]. Though several studies have tried to shed light to the precise pathogenic mechanism(s), the results are still not definitive [70,71,72] except for aseptic meningitis, a disease that seems to be directly caused by *Mycoplasma pneumoniae*. In several cases, CSF (cerebrospinal fluid) analysis has led to the identification of *Mycoplasma pneumoniae* DNA and to increased IL-6 and IL-8 concentrations. However, so far *Mycoplasma pneumoniae* antigens have never been detected in the CNS [73,74]. Similar pathogenetic mechanisms can be supposed for early-onset CNS disease related to *Mycoplasma pneumonia*, since concentrations of interleukin IL-6, IL-8, IL-18, interferon (INF)-g, tumor necrosis factor (TNF)-α, and transforming growth factor (TGF)-β1 are increased in serum of CSF samples from patients with several CNS manifestations during acute *Mycoplasma pneumoniae* infection [75]. Unfortunately, *Mycoplasma pneumoniae* DNA and cytokines could not be detected in the CSF of all cases of early-onset disease, highlighting the difficulty of the identification of the true pathogenetic mechanisms of a *Mycoplasma pneumoniae*-related neuropathy.

**Figure 1:**
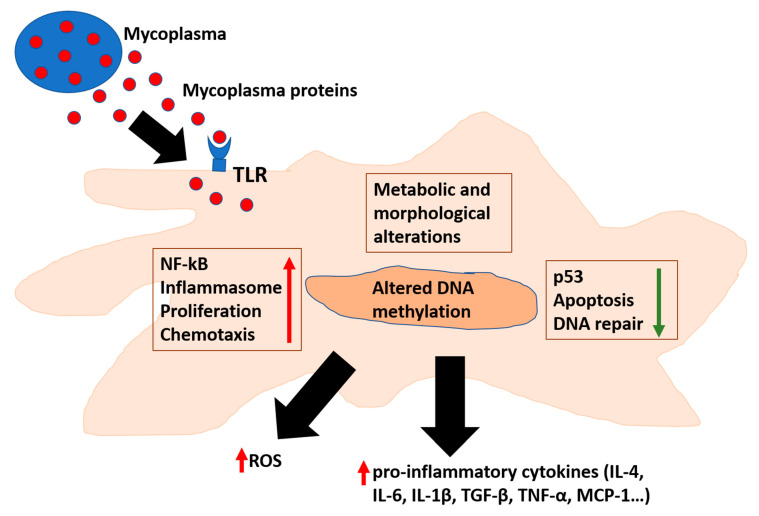
Mycoplasmas affect cellular pathways involved in inflammation and cellular transformation. Mycoplasmas’ proteins interact with TLR or enter the cells, where they can alter several pathways responsible for inflammation and DNA repair. In addition, affecting methylation of cellular DNA results in alteration of cellular epigenetic landscape. TLR: Toll Like Receptor; ROS: Reactive Oxygen Species. TGF: Transforming Growth Factor; TNF: Tumor Necrosis Factor; and MCP-Monocyte Chemoattractant Protein.

Several data have indicated *Mycoplasma fermentans* pathogenicity [28]. Indeed, *Mycoplasma fermentans* is linked to several chronic inflammatory diseases, in particular with arthritis [51]. It has also been suggested as a co-factor in AIDS disease progression [6,76]. When Mycoplasmas’ level is low, it triggers no symptoms for humans and animals [1]. However, upon a certain threshold of replication, inflammation is triggered [77] (Figure 1). The most important mechanism that triggers the immune response is the binding of *Mycoplasma* proteins to pattern-recognition receptors (PRRs)—Toll-like receptors (TLRs) and NOD-like (nucleotide-binding and oligomerization domain) receptors [32,34]. A series of cellular pathways are then engaged, and consequently a complex cascade of events determines the specificity of the immune response. TLRs 1, 2, 4, and 6 were found to bind bacterial LPS [36,37]. However, Mollicutes lack typical bacterial PAMPs (e.g., lipoteichoic acid, flagellin, and some lipopolysaccharides) and consequently the exact molecular mechanisms of Mycoplasmas’ recognition by the cells of the immune system is the subjects of several researches for its pathogenic implications.

To this regard, a protein able to bind TLRs is the macrophage-activating lipopeptide-2 (MALP-2) from *Mycoplasma fermentans* [38,39,78]. Upon binding, nuclear factor NF-kB [79] is activated and induces the expression of pro-inflammatory mediators, such as TNF-α (tumor necrosis factor-α), IL-6 (interleukin 6), MIP-1β (macrophage inflammatory protein-1β), GRO-α (growth-regulated oncogene-α), MCP-1 (monocyte chemoattractant protein-1), MIP-1α (macrophage inflammatory protein-1α) [42], CXCL13 (chemokine CXCL13), CXL14 (chemokine CXL14), RANTES (Regulated-on-Activation-Normal-T-cell-Expressed-and-Secreted chemokine) [43], and MIP-2 (macrophage inflammatory protein-2) in monocytes [44]. *Mycoplasma fermentans* infection of monocyte/macrophages increase also MMP-12 levels, a metalloproteinase which is both a pro-inflammatory molecule and necessary for Monocyte Chemoattractant Protein-1 (MCP-1) cleavage into its active form [80]. MCP-1 is involved in monocyte recruitment to the site of infection. All together, these data indicate an evolutionarily conserved nature of the mycoplasmal ligands able to elicit the same cellular signaling response. Of note, individual lipopeptides from Mycoplasmas can induce inflammation, separated from the whole microorganism, pointing to a possible paracrine effect on cells [81].

While the presence of high levels of Mycoplasmas and increased levels of inflammation can easily explain their pathogenicity, in some cases the mechanisms underlying their negative effects are not very clear. An example is chronic obstructive pulmonary disease (COPD), which in its two pathological manifestations (chronic bronchitis and emphysema) is an increasing cause of morbidity and mortality (130,000 death worldwide). Long-term exposure to irritants (mainly tobacco smoking and air pollutants) triggers an inflammatory response in the lungs, resulting in narrowing of the small airways, breakdown of lung tissue and progressive alveolar destruction (emphysema), and onset of symptoms like dyspnea, cough, and sputum production [82]. Although respiratory symptoms are the hallmarks of COPD, non-pulmonary manifestations occur frequently; thus, increasing risk of significant cardiovascular, endocrine, and musculoskeletal comorbidities [83]. These non-pulmonary manifestations are most likely mediated by immune-dysfunction initiated by inflammatory processes that are initially triggered within the lungs and propagate systemically both causing and accentuating comorbidities [84]. To this regard, increased levels of circulating inflammatory biomarkers observed in COPD patients are potential mediators of these systemic effects [85]. In addition, COPD patients also have significantly higher levels of circulating functional T-regulatory cells (Tregs), myeloid-derived suppressor (MDSC) cells, and exhausted programmed Death (PD) 1 + cells, which contribute to effector T-cell dysfunction and reduce their ability to fight infections [86,87]. The characterization of lung microbiota lead to the discovery of a significant reduction in diversity, compared to microbiota observed in healthy persons. In particular, in COPD patients the composition of microbiota seems to be restricted to phyla which include potentially pathogenic microorganisms, such as *Mycoplasma pneumoniae* [88,89,90], which is also associated with acute exacerbation [91,92].

### 2.2. Mycoplasmas Causing Diseases in Animals

Regarding their role as pathogenic agents for animals, we mention first *Mycoplasma mycoides* subsp. *mycoides* Small Colony (SC), responsible for bovine pleuropneumonia (CBPP), which among the several *Mycoplasma* species is arguably the most pathogenic. A massive inflammatory reaction predominantly involving the lungs of the infected host is the most important pathological manifestation of this *Mycoplasma* species, causing lung consolidation and leading to respiratory distress and death in 25–35% of the cases. In the remaining majority of infected animals, CBPP assumes a chronic form, with recovery from the acute stage of the disease but where the animal host remains a potential carrier and consequently a reservoir of *Mycoplasma mycoides* subsp. *mycoides* SC. The CD4 Th1-like T-cell response to the pathogen was observed in animals recovered from disease over the entire duration of the experiments lasting for over five months. This contrasted with the observation that symptomatic CBPP progression correlated with PBMCs reduced capacity to produce interferon in animals that developed an acute disease [93,94]. Morphological changes in mononuclear cells from bovine PBMCs were observed in vitro upon infection with *Mycoplasma mycoides* subsp. *mycoides* SC. Such changes included increased cell granularity and reduced cell size. The observation that heat inactivated *Mycoplasma* was unable to induce the same changes, further highlights the requirement for viable *Mycoplasma mycoides* subsp. *mycoides* SC and productive infection. These changes eventually lead to a cytopathic effect responsible for the apoptosis of the mononuclear cells. This effect was minimal when *Mycoplasma* free culture supernatants were used, indicating that the responsible protein was probably released by the infected cells upon infection [95]. Among the possible cytokines potentially responsible for the cytopathic effect, it was demonstrated in a different study that *Mycoplasma mycoides* subsp. *mycoides* SC strains can induce TNF-α production in bovine alveolar macrophages [96]. Other factors able to cause cell death by damaging DNA are the reactive oxygen species (ROS), that are produced by *Mycoplasma mycoides* infection *through* the metabolism of glycerol upon leukocytes activation. The proposed mechanism involves: (i) *Mycoplasma mycoides* adhesion to the surface of the cells, (ii) activation of TLRs and consequent promotion of their respiratory burst; and (iii) production and translocation of increased ROS amounts within the phagocytic cell of the host; thus, causing an irreparable damage to the cell membranes. The resulting inflammatory reaction could thus contribute to changes in lung morphology and to function impairment [97].

Another *Mycoplasma*, *Mycoplasma capricolum* subsp. Capripneumoniae, is highly pathogenic when localized in the caprine mammary gland. It causes acute mastitis, initially purulent. Massive fibrosis ensues after a phase of infiltration of lymphonuclear cells, followed by fibroplasia in the interacinar tissue [98]. *Mycoplasma capricolum* infection is also responsible for a disease in the goats (contagious caprine pleuropneumonia—CCPP), where considerable inflammatory infiltrates are detected in the injured lungs during CCPP development and lung damage is caused by increased IL-17 production and consequent accumulation of neutrophil within the alveoli [99].

We also mention *Mycoplasma agalactiae*, responsible for the agalactia syndrome in sheep and goats, a contagious disease that produces considerable economic losses worldwide. The primary mechanism(s) whereby *Mycoplasma agalactiae* infection damages the host cells are not completely clear, and the most credited hypothesis considers the host immune response as the major responsible for the excessive inflammation and consequent tissue destruction. To this regard, in vitro infection of HeLa cells resulted in some morphological changes, namely cell elongation, cytoplasm shrinkage, and membrane blebbing. These changes, together with chromatin condensation and increased caspase-3 activation indicate an apoptosis-like phenomenon leading to reduced cell viability and increased cell lysis [100]. Additionally, it was observed an association with *Mycoplasma agalactiae* antigen and production of IL-10, IFN-γ, IL-4, and TNF-α in an experimental in vitro model consisting of inflammatory cells of mammary tissues from goats infected with *Mycoplasma agalactiae*, [101]. Finally, sheep infected with *Mycoplasma agalactiae* showed prolonged depletion of peripheral CD3^+^CD4^+^ and CD3^+^CD8^+^ cells, possibly due to organ infiltration. Real-time PCR assay allowed the detection of the infectious agent in different areas (ear, nose, and milk) up to 50 days post infection [102].

Finally, MALP-2 from some strains of *Mycoplasma gallisepticum* induces the expression of TNF-α, IL-6, and MIP-1β in chickens [43]. Interestingly, it was observed a differential role of TLR2-2 and TLR6 in *Mycoplasma gallisepticum*-infected DF-1 cells and chicken embryos [103].

**Table 1:** Association between several species of *Mycoplasma*, diseases, and proposed mechanism(s) of inflammation.

*Mycoplasma* Types	Diseases and Proposed Mechanism(s) of Inflammation
	Human-Associated Mycoplasmas
Mycoplasmas (general)	Respiratory diseases [55], Urogenital diseases [104], Rheumatoid Arthritis [52], Fibromyalgia [105,106], and Neurological diseases [107,108]. *Mycoplasma* proteins bind to pattern-recognition receptors (PRRs)—Toll-like receptors (TLRs) and NOD-like (nucleotide-binding and oligomerization domain) receptors [32,34,36,37].
*Mycoplasma genitalium*	Urogenital infections [47]. Adhesion to epithelial cells promotes acute inflammation via triggering of innate immune sensors expressed on the cells’ surface. Activation of pro-inflammatory signals ultimately results in recruitment of leucocytes to the infection site. The recombinant C-terminal portion of the immunogenic protein MG309 (rMG309c) activates NF-κB via TLR2/6 in genital epithelial cells (EC), which in turn secreted proinflammatory cytokines, including interleukin-6 (IL-6) and IL-8 [109,110].
*Mycoplasma pneumoniae*	Respiratory diseases [55]. Different adhesins and accessory adhesion proteins mediates the crucial initial step of cytoadherence to respiratory tract epithelium, Subsequently, several mechanisms, namely intracellular localization, direct cytotoxicity and toll-like receptors (TLRs)-mediated activation of the inflammatory cascade cause tissue injury mediated by such cytokines. Infection is associated with acute exacerbation of COPD [91,92], and COPD patients also have significantly higher levels of circulating functional T-regulatory cells (Tregs), myeloid-derived suppressor (MDSC) cells and exhausted programmed Death (PD) 1 + cells, which contribute to effector T-cell dysfunction and reduce their ability to fight infections [86,87]. In infected mice is observed a dysregulated *Mycoplasma pneumoniae*-derived immune response in lung [81,88,89,90]. *Mycoplasma pneumoniae* also is responsible for Community-Acquired Respiratory Distress Syndrome toxin (CARDS toxin), which activates adenosine diphosphate (ADP) ribosylation and inflammasome, causing airway inflammation. [111]. Inflammatory mediators, namely interleukin IL-6, IL-8, IL-18, interferon (INF)-g, tumor necrosis factor (TNF)-α, and transforming growth factor (TGF)-β 1 are increased in serum of CNS [54].
*Mycoplasma hominis*	Urogenital infections (pelvic inflammatory diseases and bacterial vaginosis) [112,113,114,115,116,117].
*Mycoplasma penetrans*	Urogenital infections [116], Autoimmune disorders: Immunoglobulin A nephropathy [118]. Secreted P40 mediates (partly) cytotoxicity upon infection of *Mycoplasma penetrans* in vitro, by inducing physiological modifications resembling apoptosis [119].
*Mycoplasma salivarium*	Septic arthritis [120,121], periodontal disease [122,123,124]. Cell membranes of *Mycoplasma salivarium* promote expression of IL-6 and IL-8 in human fibroblasts through stimulation of protein kinase C (PKC) in Gin-1 cells, a human gingival fibroblast cell line [125].
*Mycoplasma fermentans*	Urogenital diseases [104], Rheumatoid Arthritis [52]. *Mycoplasma fermentans* increases the secretion of macrophage-activating lipopeptide-2 (MALP-2) [38,39,78], TNF-α (tumor necrosis factor-α), IL-6 (interleukin 6), MIP-1β (macrophage inflammatory protein-1β), GRO-α (growth-regulated oncogene-α), MCP-1 (monocyte chemoattractant protein-1), MIP-1α (macrophage inflammatory protein-1α) [39,42,79], CXCL13 (chemokine CXCL13), CXL14 (chemokine CXL14), RANTES (Regulated-on-Activation-Normal-T-cell-Expressed-and-Secreted chemokine) [43], MCP-1 (monocyte chemoattractant protein-1), MIP-1α (macrophage inflammatory protein-1α) [42]. *Mycoplasma fermentans* infection of monocyte/macrophages increases also MMP-12 levels, a metalloproteinase which is both a pro-inflammatory molecule and also necessary for the cleavage of Monocyte Chemoattractant Protein-1 (MCP-1) into its active form [80]
	**Animal-Associated Mycoplasmas**
*Mycoplasma mycoides*	In bovine hosts, it is observed: increased production of TNF-α in alveolar macrophages (cattle) [96]; induction of morphological changes in mononuclear cells [95]; induction of ROS [97].
*Mycoplasma capricolum*	Contagious caprine pleuropneumonia (CCPP) is associated with increased IL-17 and neutrophil accumulation, leading to lung injury [99]
*Mycoplasma agalactiae*	Infection of HeLa cells lead to morphological changes including membrane blebbing, which together with increased caspase-3 cleavage activity indicated an apoptosis-like phenomenon [100]. An in vitro model consisting of inflammatory cells of mammary tissues from goats infected with *Mycoplasma agalactiae* demonstrated an association with *Mycoplasma* antigen(s) and production of IL-10, IFN-γ, IL-4, and TNF-α [101]
*Mycoplasma gallisepticum*	MALP-2 from some strains of *Mycoplasma gallisepticum* induces the expression of TNF-α, IL-6, and MIP-1β in chickens [43]. Interestingly, it was observed a differential role of TLR2-2 and TLR6 in *Mycoplasma gallisepticum*-infected DF-1 cells and chicken embryos [103].

## 3. Mycoplasmas and Cancer

Definitive establishment of the causal correlation between *Helicobacter pylori* and gastric cancer provided the first demonstration that bacteria can cause cancer [126]. Since then, studies of the human microbiome have elucidated an array of complex interactions between prokaryotes and their hosts [127]. Recent examples of studies in human patients highlighted an association between *Fusobacterium nucleatum* and colorectal cancer [128,129,130,131,132,133,134], and between Mycoplasmas and prostate and colorectal cancer, oral carcinoma associated with Fanconi anemia [123], as well as non-Hodgkin’s lymphoma (NHL) in HIV-seropositive subjects [123,135,136,137,138,139]. These data strongly support them as leading bacterial candidates with oncogenic properties (Figure 1).

Indeed, several data obtained by using mouse models with particularly mutated genes, or in vivo experiments carried on with cancer-inducing agents, showed that tumor formation is reduced when the mice colonies are grown and kept in a germ-free environment [140,141].

The precise pathogen–cancer relationships of a number of bacteria, including Mycoplasmas, remain largely elusive. In particular, we note that some bacteria are able to establish persistent, chronic infection by invading the host’s cell and remaining undetected by the immune system for a long period of time. They produce proteins that interfere and alter the function of important cellular pathways like cell cycle control, apoptosis, DNA repair. This, linked to the ability of these pathogens to induce substances able to increase DNA damage may increase abnormal cell growth and transformation [142,143].

A number of studies have established a firm link between chronic inflammation, tumor progression and p53, which, undoubtedly, is the most important tumor suppressor protein in humans, given its central role in preserving genome stability [144,145]. NF-κB reduces the activities of p53 and the mutual regulation between antiapoptotic NF-κB and proapoptotic p53 is one of the major determinant of a cell’s fate [146]. In fact, genetic or pharmacological inhibition of constitutively active NF-κB in different tumor cell lines leads to the activation of p53 function and tumor cell death via p53-dependent apoptosis [147]. Given that inflammation can reduce the activity of p53, it is possible that chronic inflammation through the activation of NF-κB reduces the activity of p53; thus, promoting cellular transformation [146].

Following DNA damage and other stress signals, low levels of cellular p53 protein increase, causing growth arrest, DNA repair, or apoptosis. Interruption of cell cycle prevents replication of damaged DNA, allowing p53 to activate the transcription of proteins involved in DNA repair. On the other hand, when this pathway is compromised the cell activates the pathways leading to apoptosis, which is the mechanism of choice to avoid proliferation of cells containing abnormal DNA [148].

For these reasons, the cellular concentration and activity of p53 must be tightly regulated, and the major regulator of p53 is Mdm2, which functions by retaining p53 in the cytoplasm and activating its degradation by the ubiquitin system [149,150,151]. Mdm2 is regulated by p53 through a feedback mechanism, and by the genes involved in growth arrest, DNA repair, and apoptosis (such as p21, Gadd45, BAX, and PUMA) [152,153].

It is well known that several strains of *Mycoplasma* suppress the transcriptional activity of p53, resulting in reduced apoptosis of damaged cells and some Mycoplasmas (notably *Mycoplasma fermentans*, *Mycoplasma penetrans,* and *Mycoplasma hyorhinis*) were reported to have oncogenic potential since they demonstrated not just accumulation of abnormalities but also phenotypic changes of the cells [154,155,156] (Table 2). Moreover, long-term *Mycoplasma* infections in cell cultures are associated with increased frequency of chromosomal instability and malignant transformation such as the lost cell-to-cell contact, the spindle morphology and the growth in multiple layers [154]. These changes were reversed when earlier cultured cells (maintained for up to six passages in vitro) were treated with three cycles of ciprofloxacin and returned to a normal growth pattern [154]. On the contrary, long-time cultured cells (for more than 18 passages) were not able to acquire their previous morphology/growth pattern when treated with the same antibiotic, demonstrating an irreversible change. These data indicate that persistent infection with *Mycoplasma* induce cellular transformation through a series of cellular events [154]. In addition, spontaneous transformation of mouse embryo fibroblasts and concomitant overexpression of the H-ras and c-myc proto-oncogenes were observed upon long-term infection with *Mycoplasma fermentans* or *Mycoplasma penetrans* [157].

Moreover, upon infection, several species of human Mycoplasmas would prevent apoptosis in 32D cells from undergoing in vitro in the absence of IL-3, indicating continuous growth even in the absence of the important IL-3 growth signaling. To this regard, it was observed that infected 32D cells gradually underwent malignant transformation after a period of 4 to 5 weeks and no longer needed the presence of either *Mycoplasma fermentans*, *Mycoplasma penetrans*, nor of IL-3 to grow. Not surprisingly, these 32D cells were able to grow independently and were highly tumorigenic upon injection into a nude mice model. Karyotyping analysis demonstrated chromosomal changes and trisomy 19 associated with malignant transformation [155].

Another potential way that Mycoplasmas have to influence cancer formation, is by deregulating expression of Bone morphogenetic protein 2 (BMP2), which is an essential growth factor and morphogen, implicated in cancer promotion and growth [158,159]. In fact, it has been shown that infection by *Mycoplasma penetrans*, *Mycoplasma fermentans,* and *Mycoplasma hominis* induces BMP2 RNA expression, as well as secretion of mature BMP2 protein, in cells that usually do not express such protein, including BEAS-2B cells (immortalized human bronchial epithelial cells), A549 cells (lung adenocarcinoma cells), plus several other cell lines of different origins (mesenchymal, epithelial, and myeloid). This increase in BMP2 expression in *Mycoplasma*-infected cells was mostly achieved by regulating RNA stability, rather than influencing the transcriptional level. Additionally, it was demonstrated that BMP2 stimulated proliferation of BEAS-2B cells transformed by chronic *Mycoplasma* infection, indicating the profound effects of *Mycoplasma* infection on BMP2-regulated pathways, including the ones involved in cell proliferation, differentiation, and apoptosis [136].

*Mycoplasma hyorhinis* expresses p37 protein on its surface, and this protein belongs to a high-affinity transport system associated with cancers in animals and humans. Indeed, p37 induces rapid expression of several genes involved in inflammation and cancer progression through TLR4 receptor triggering in fibroblasts. As cancer associated fibroblasts favor growth, invasion, and metastasis by regulation of tumor-related inflammation, p37 may influence cancer development by inducing expression of pro-inflammatory genes [160]. To this regard, p37 increased migration in a transwell (Matrigel) assay of human gastric carcinoma (AGS) cells by inducing the phosphorylation of epidermal growth factor receptor (EGFR) and extracellular signal-regulated kinase and the activity of matrix metalloproteinase-2 (MMP-2) [161].These results indicate that p37 may be able to promote invasion by upregulating the activity of MMP-2; thus, causing EGFR phosphorylation and increasing tumor metastasis upon *Mycoplasma hyorhinis* infection. Additional type of cancers that seem to be influenced by p37 are PC-3 and DU145 (two prostate cell lines), since treatment with p37 increased invasivity and migratory ability, as demonstrated by a Matrigel-based assay [161,162]. To this regard, it was observed a significant nuclear enlargement, denoting active, anaplastic cells following incubation with recombinant p37. Microarray analysis of p37-treated cells allowed to identify eight clusters of differentially expressed genes broadly divided into three groups. The most represented categories of functional genes were composed by signal transduction, cell cycle, and metabolic factors [163]. Treatment with p37 also affected Ficoll-separated human peripheral blood mononuclear cells (PBMCs), increasing the expression of *tumor necrosis factor α* (*TNFα)* gene transcription and the secretion of TNFα [160]. This also indicates that p37 and its regulated molecules could be potentially targeted for anti-cancer intervention [161].

Expanding on these studies, it was also shown that *Mycoplasma fermentans*, is able to influence the expression of hundreds of genes in cultured human cells; thus, affecting many pathways. This regulation involved increased or reduced expression of many cytokines, stress-response genes, transport proteins, receptors, ion channels, growth factors, oxidases, tumor suppressors, and oncogene during a two-stage process; a reversible one, when the transformation process can be stopped by eradicating the *Mycoplasma*, and an irreversible phase [138].

Further in vivo experiments demonstrated the oncogenic potential of *Mycoplasma penetrans* in immunocompromised settings. Upon infection, mice immunosuppressed with cyclophosphamide had lower expression of p53 and p21 and higher expression of H-ras in gastric mucosa, compared to the uninfected animals. Moreover, NF-κB p65 subunit and TNF-α expression increased in infected mice. On the other hand, Bax expression was lower while Bcl-2 expression was higher. These data demonstrate that *Mycoplasma* infection reduces the levels of several oncogenes in the gastric mucosa of immunodeficient mice, and this could potentially facilitate the malignant transformation of these cells [164].

**Table 2:** Association between several species of Mycoplasmas, cancer(s), and proposed mechanisms of cellular transformation.

*Mycoplasma* Types	Cancer(s) and Proposed Mechanisms of Cellular Transformation
*Mycoplasma fermentans* and *Mycoplasma penetrans*	Increased expression of BMP2 upon infection [136]. *Mycoplasma fermentans and Mycoplasma penetrans* infection induced malignant transformation of 32D cells (including autonomous growth in IL-3-conditions). After a few weeks, the presence of Mycoplasmas was no longer needed for autonomous growth of the cells. Transformed 32D cells were able to form tumors when injected into nude mice. Karyotyping analysis showed chromosomal abnormalities, including trisomy 19 associated with malignant transformation [154,155,156]. Several mechanisms account for their potential cell-transforming effect: induction of genetic instability, alterations in metabolism, changes in the expression of many genes, in particular growth factors, tumor suppressors and oncogenes [164]
*Mycoplasma genitalium*	*Infection* promoted a malignant phenotype in benign human prostate cells (BPH-1), as assessed by in vitro and in vivo assays showing anchorage-independent growth, greater percentage of migrating cells with increased invasive capacity, generation of xenograft tumors in athymic mice and accumulation of chromosomal aberrations and polysomy [137].
*Mycoplasma hominis*	*Infection* promoted a malignant phenotype in benign human prostate cells (BPH-1), similar to *Mycoplasma genitalium* [137]. Higher titers of antibodies against *Mycoplasma hominis* were observed in prostate cancer positive patients, together with higher average PSA levels [139]. Infection promoted expression of BMP2, similar to *Mycoplasma penetrans* and *Mycoplasma fermentans* [136].
*Mycoplasma hyorhinis*	p37 seems to be the major determinant involved in events potentially leading to cell transformation: (1) it induces the expression of genes implicated in inflammation and cancer progression in fibroblasts, indicating that cancer associated fibroblasts may facilitate growth, invasion and metastasis by regulating tumor associated inflammation [160]; (2) when added to human gastric carcinoma cells (AGS) increased the migration in a transwell (Matrigel) assay, by promoting phosphorylation of epidermal growth factor receptor (EGFR) and extracellular signal-regulated kinase and the activity of matrix metalloproteinase-2 (MMP-2) [161]; (3) it induces significant nuclear enlargement, indicating the generation of active, anaplastic cells and promoted the migratory capacity of both PC-3 and DU145 cells [162,163]; and (4) microarray analysis of p37-treated cells identified eight gene expression clusters classified into three groups, with cell cycle, signal transduction and metabolic factors among the most represented genes [163].
*Mycoplasma penetrans*	Infection in vivo is associated with lower expression of p53 and p21 and higher H-ras expression in gastric mucosa. Moreover, expression of NF-κB p65 subunit increased together with TNF-α expression are observed, and Bax expression was lower while Bcl-2 expression was higher. These data indicate that persistent infection is associated with aberrant expression of multiple proto-oncogenes in gastric mucosa of immunodeficient mice suggesting its potential influence on malignant transformation. [164].
*Mycoplasma salivarium*	Possible role in oral cancer [123,165].
*Mycoplasma fermentans*	*Mycoplasma fermentans* reduced activity and expression of Topo I [166]. Reduction of p53 activity [167,168], reduction of PARP-1 activity [168,169]
*Mycoplasma arginini*	infection in vivo resulted in suppression of p53, activation of NF-kB and increased Ras mutagenic effects, similar to *Mycoplasma penetrans* [167].

Additionally, *Mycoplasma* infection reduced activation of p53 with a constitutive activation of NF-κB in cells infected with *Mycoplasma*, further highlighting its effects of on these important regulatory pathways [167]. This altered expression was consistent with many human tumors. Thus, infected cells were able to evade apoptosis by inhibiting p53 [167,170,171]. Though the responsible *Mycoplasma* protein was not identified, more recent works from our group point to a *Mycoplasma* chaperon protein, DnaK, a chaperone protein belonging to the HSP70 family, as responsible for reduction of pathways linked to DNA repair, cell cycle control and apoptosis [168]. In particular, following the isolation of a strain of *Mycoplasma fermentans* able to induce lymphoma in a severe combined immuno-deficient (SCID) mouse model [170,171,172], we characterized the molecular mechanisms in vitro. We showed that this *Mycoplasma* DnaK, co-immunoprecipitates with USP10 (ubiquitin carboxyl-terminal hydrolase 10), a key p53 regulator [173], and impairs p53-dependent anti-cancer activities [168].

We showed that the binding of DnaK to PARP1, which recognizes DNA breaks and participates in DNA repair [174,175,176,177], reduces its activity and, following recognition of damaged DNA, PARylation of certain proteins of very high MW is greatly reduced (> 150 KDa), while it seems it only marginally affects proteins between 100–150 KDa [168,169]. We could abundantly find sequences of *Mycoplasma* DnaK early in infected mice, while only a low amount of copy number was found in primary and secondary tumors, pointing to a “hit and run/hide” mechanism [168]. Given the fact that infections with certain Mycoplasmas lead to ROS production [49,178], and ROS can cause direct damage to DNA, our data provide a molecular link between a *Mycoplasma* protein, DnaK, and cellular transformation.

Further studies linking *Mycoplasma* to carcinogenesis are illustrated by its involvement in changes in DNA methylation pattern. DNA methylation (that is the conversion of cytosine to 5-methylcytosine) is an essential element in transcriptional regulation and is one of the major epigenetic mechanisms. Many stresses or DNA damage can in fact interfere with the ability of DNA to be methylated at CpG dinucleotides by DNA-methyltransferases (DNA-MTases) [179]. When specific *Mycoplasma* MTases were expressed in human cell lines, their translocation to the nucleus has been observed. The result was a change of the human genome methylation landscape because these bacterial enzymes methylated cytosines within the respective CG and GATC sites in human genomic DNA, resulting in the stimulation of pro-oncogenic pathways [180].

Additional reports have strongly suggested a role for *Mycoplasma* in cellular transformation and the search for the link between *Mycoplasma* and cancer is currently actively being investigated. To this regard, many studies demonstrated the effects of *Mycoplasma* on cell lines by showing that *Mycoplasma* may facilitate tumorigenesis, for example in oral tissues [165], in human prostate cells [137,139] in gastric carcinoma cells [181] and cervical cells both in vitro [182].

In vivo, several studies reported the isolation of *Mycoplasma* species in various neoplastic tissues and body fluids, and in particular Mycoplasmas have been found in precancerous lesions as well as in malignant tissues from patients with stomach, colon, ovarian and lung cancers, and hepatocellular carcinoma [142,183,184], though no direct causal relationship with cellular transformation has been demonstrated so far. Nonetheless, all the outlined studies and properties of Mycoplasmas strongly suggest that these agents act as cancer-promoting factors.

## 4. Conclusions

Several different bacteria have been associated with human cancers. A widespread and concerted scientific effort is ongoing to identify potentially responsible bacteria and characterize the molecular mechanism(s). While *Helicobacter pylori* so far is the only one with clear data to support causality [126], studies of other bacteria including Mycoplasmas [123,135,139] strongly support the idea that they too have oncogenic properties. Experimental results have demonstrated the role of Mycoplasmas in increasing inflammation and associated them to cancer initiation.

Although it seems plausible that accumulation of DNA-damage and inhibition of p53-activities play a major role in driving transformation, molecular mechanisms whereby these bacteria dysregulate cellular pathways and eventually result in cellular transformation are still largely unknown. By linking inflammation, DNA damage and reduction of p53 activity, it may be possible to formulate a hypothesis to better define the role of Mycoplasmas in causing cellular transformation and disease.
